# Draft genome assembly of the biofuel grass crop *Miscanthus sacchariflorus*

**DOI:** 10.12688/f1000research.44714.1

**Published:** 2021-01-18

**Authors:** Jose De Vega, Iain Donnison, Sarah Dyer, Kerrie Farrar

**Affiliations:** 1 Earlham Institute, Norwich, NR4 7UZ, UK; 2 Institute of Biological, Environmental & Rural Sciences (IBERS) - Aberystwyth University, Aberystwyth, SY23 3EE, UK

**Keywords:** Miscanthus, biofuel, C4, assembly, annotation

## Abstract

*Miscanthus sacchariflorus* (Maxim.) Hack. is a highly productive C4 perennial rhizomatous biofuel grass crop. *M. sacchariflorus* is among the most widely distributed species in the genus, particularly at cold northern latitudes, and is one of the progenitor species of the commercial *M. × giganteus *genotypes. We generated a 2.54 Gb whole-genome assembly of the diploid *M. sacchariflorus* cv. “Robustus 297” genotype, which represented ~59% of the expected total genome size. We later anchored this assembly using the chromosomes from the *M. sinensis* genome to generate a second assembly with improved contiguity. We annotated 86,767 and 69,049 protein-coding genes in the unanchored and anchored assemblies, respectively. We estimated our assemblies included ~85% of the *M. sacchariflorus* genes based on homology and core markers. The utility of the new reference for genomic studies was evidenced by a 99% alignment rate of the RNA-seq reads from the same genotype.  The raw data, unanchored and anchored assemblies, and respective gene annotations are publicly available.

## Introduction

*Miscanthus* is a genus of C4 perennial rhizomatous grasses native to East Asia and Oceania, and naturally adapted to a wide range of climate zones and land types. *Miscanthus sacchariflorus* is among the most widely distributed species within the genus. It originated in the Yellow Sea region of China and can be predominantly found in cool latitudes of East Asia with varying ploidy ^1^. *M. sacchariflorus* occurs in both diploid (2n = 38) and tetraploid (2n = 76) forms, where tetraploid *M. sacchariflorus* genotypes originated by autopolyploidy ^2^. *M. sacchariflorus* probably has the greatest winter hardiness among all the Saccharinae ^3^.

Natural interspecific *Miscanthus* hybrids are commonly observed, even between individuals of different ploidy. For example, introgression of *M. sacchariflorus* is often found among cultivated European *M. sinensis* ecotypes ^1, 4^. Furthermore, *M. x giganteus*, a sterile triploid hybrid resulting from the hybridization between *M. sinensis* and *M. sacchariflorus*, is the predominant commercially grown species owing to its high biomass productivity and low chemical input requirements. The common occurrence of hybridization events and variable ploidy are challenging to the improvement of these bioenergy grasses and increase the need for genomic resources from different *Miscanthus* species. A chromosomal-scale reference genome using a doubled-haploid *M. sinensis* line was recently published ^4^.

We assembled, annotated and validated a draft genome from the diploid *M. sacchariflorus* cv. “Robustus 297” genotype, as well as generating rhizome, stem and leaf RNA-Seq data from the same genotype. This dataset was previously used to verify that both *M. sinensis* and *M. sacchariflorus* share the same A/B ancestral tetraploidy ^4^. Here, we present the first draft genome of *M. sacchariflorus*, the second *Miscanthus* genome available after *M. sinensis* ^4^.

## Methods

### Plant materials and sequencing

DNA was extracted from leaves from the diploid *M. sacchariflorus* cv. “Robustus 297” genotype (Biosample SAMN08580354) using the Qiagen DNeasy kit. RNA was also extracted from leaf, stem and root tissues from the same plant. All samples were taken from a plant grown from seed in trays in a glasshouse in 2009. This genotype is established and used in breeding at IBERS (Wales, UK). The RNA-seq libraries were deposited as part of previous work in the BioProject PRJNA639832.

### Whole genome sequencing and assembly

We obtained ~5.86e9 pairs of 100 bp paired-end reads from an Illumina paired-end library with a 560 bp insert-size that was sequenced on Illumina HiSeq 2500 machines in rapid run mode by the Earlham Institute. This represents approximately 50X coverage of the heterozygous content and 100X coverage of the homozygous content of the genome. Read quality was assessed, and contaminants and adaptors removed using Kontaminant ^5^. These paired-end short-reads were assembled into 17M contigs with a total length of 3.27 Gb using ABySS ^6^ version 1.5.1, with default options and a kmer size of 71.

We obtained ~141.1e6 pairs of reads from a Nextera 150 bp mate-pair library with approximately 7 Kb insert-size, which was used for scaffolding the previous contigs together with the paired-end reads, using SSPACE ^7^ without “extension” step. Nextera mate-pair reads were required to include a fragment of the adaptor to be used in the scaffolding step ^5^, and we filtered out sequences shorter than 500 bp. We obtained 589K scaffolds, a total length of 2.54 Gb with an N50 of 10.2 Kb. This whole-genome assembly was denominated “Msac_v2” and is deposited at NCBI in BioProject PRJNA679435.

### Gene model and functional annotations

Our gene structure annotation pipeline ^8^ used five sources of evidence that were provided to AUGUSTUS ^9^ (version 2.7) for gene annotation: (1) Repetitive and low complexity regions of the scaffolds identified using RepeatMasker ^10^ (version open-4.0.5) based on homology with the RepBase ^11^ public database (Release 20140131) and a new database of repeat elements identified in the assembly with RepeatModeler ^12^. The repeats annotation was deposited in Zenodo (See data availability); (2) exon-intron junctions identified by Tophat ^13^ (version 2.1.0); (3) *de novo* and genome-guided *ab initio* transcripts assembled with Trinity ^14^ (version 2.6.5 )and Cufflinks ^15^ (version 2.2.1) from RNA-Seq reads obtained from several tissues from the same genotype; (4) *ab initio* gene models predicted by SNAP ^16^ (version 29-11-2013) and GeneID ^17^ (version 1.4.4); and (5) homology-based alignments of transcripts and proteins from *Miscanthus sinensis* and maize using Exonerate ^18^ with a minimal identity of 0.7 and coverage of 0.7. Finally, AUGUSTUS ^9^ was run with the options “genemodel=complete” and “alternatives-from-evidence=true” to ensure that the predicted genes were compatible with all the previous provided evidence.

For the functional annotation of these predicted genes, translated gene sequences were compared with the NCBI non-redundant (nr 20170116) proteins and EBI’s InterPro (version 5.22.61) databases, and the results were imported into Blast2GO ^19^ to annotate the GO and GO slim terms, enzymatic protein codes and KEGG pathways. A similar GO annotation from translated gene sequences can be done with eggNOG-mapper ^20^. These functional descriptors were deposited in Zenodo (See *Underlying data*).

### Anchoring the whole genome assembly using the Miscanthus sinensis reference

To improve the genome contiguity, we anchored our *M. sacchariflorus* scaffolds to the Miscanthus sinensis genome ^4^. However, no nucleotide content from *M. sinensis* was incorporated in the *M. sacchariflorus* assemblies.

Firstly, scaffolds longer than 2 kbps from the whole genome assembly “Msac_v2” were scaffolded again using SSPACE ^7^ and the *M. sinensis* mate-pairs reads, the gaps between scaffolds were filled in with Ns. This new whole-genome assembly was denominated “Msac_v3”, and was deposited at NCBI in Bioproject PRJNA435476, under the GenBank accession GCA_002993905. It contains 137,916 scaffolds for a total of 2.074 Gb with an N50 of 25.6 Kbps. The gene annotation was projected to the “Msac_v3” assembly using PASA ^21^ (version 2.0.1): genes were aligned to the new assembly using GMAP, requiring a minimum identity of 0.85 and coverage of 0.55, and later validated using the default parameters in PASA.

Finally, we obtained the chromosomal position in the *M. sinensis* chromosomes of the scaffolds from the “Msac_v3” assembly. Using Satsuma2 ^22^ (version untagged-330e3341a1151a978b37), we identified every perfect-identify match between both assemblies (3,635,504 matches in total). The coordinates of these matches in BED 8 format were used as input to the “OrderOrientBySynteny” script from Satsuma2, which identifies the best chromosomal position for each scaffold. These position coordinates are available as an AGP file as part of GCA_002993905, which anchors our final whole-genome assembly to 19 chromosomes (accessions CM00959 to CM009609 in NCBI).

### Completeness assessment

RNA-seq cleaned reads from each tissue were independently aligned to both assembly versions using STAR ^23^ (version 2.6.0c). BUSCO ^24^ (version 4.1.4) was used to assess completeness with the single-copy orthologs database for green plants (Viridiplantae, version 2020-09-10). Orthologs were identified using Orthofinder2 ^25^ (version 2.3.12) with default parameters and the option “-msa”, which directly provided comprehensive statistics comparing the provided proteomes. All the proteomes from the other species used ( Table 1) were downloaded from Phytozome (v7.1 DOE-JGI). Genomes were aligned using Minimap2 ^26^ (version 2.17) with the “asm10” parameter for related genomes, secondary alignments (tp:A:S) filtered out, and results visualised using dotPlotly ^27^ (Github version, latest updated on 4 May 2018).

**Table 1.** Completeness statistics of the unanchored and anchored *M. sacchariflorus* whole-genome assemblies in comparison to the *M. sinensis* reference.

	*Msac_v2* (unanchored)	*Msac_v3* (anchored by *M. sinensis*)	Reference: *M. sinensis ^4^*.
NCBI bioproject	PRJNA679435	PRJNA435476 (GCA_002993905)	v.7.1 from Phytozome
Length	2.539 Gb	2.074 Gb	1.68 Gbps
Scaffolds	588,758 scaffolds	137,931 scaffolds *	19 Chrs and 14,414 scaffolds
N20	25.39 Kbps	62.61 Kbps	146.1 Mbps
N50	10.25 Kbps	25.63 Kbps	88.51 Mbps
N80	2.79 Kbps	9.42 Kbps	75.06 Mbps
Max	378.48 Kbps	458.83 Kbps	160.9 Mbps
ANNOTATION	*Msac_v2*	*Msac_v3*	*M. sinensis*
Gene models	81,431	68,578	67,967
Proteins	86,767	68,578 **	67,789
BUSCO	*Msac_v2*	*Msac_v3*	*M. sinensis*
Complete	55.5% (48% in single copy)	59.8% (50.4% in single copy)	97.6% (36.2% in single copy)
Fragmented	32.2%	26.4%	1.6%
Missing	12.3%	13.6%	0.8%
RNA MAPPING	*Msac_v2*	*Msac_v3*	*M. sinensis*
Reads mapping in the genome once (root, stem and leaf)	76.2% 76.4% 78.8%	75% 76.7% 78.1%	78.8% *** 83.5% 82.5%
Reads mapping in the genome multiple times (root, stem and leaf)	22.5% 23% 20.7%	19.5% 18.8% 17.3%	19.7% *** 15.5% 16.6%

*15 scaffolds from plastids were discarded during the deposit in NCBI resulting in 137,916 scaffolds. ** Only the longest transcript was considered in each projected locus. *** Cross-species alignments.

## Results

We produced two whole-genome assemblies for *M. sacchariflorus* that we named “Msac_v2” and “Msac_v3”, with total lengths of 2.54 Gbps and 2.074 Gbps, respectively ( Table 1). The difference in size is mainly a result of filtering 402 Mb from sequences under 2 kb in the latter before anchoring to the *M. sinensis* genome. Our “Msac_v2” assembly covered ~59 % of *M. sacchariflorus* genome size, which is estimated to be 4.3 Gb ^28^. Approximately 40% of the assembly was composed by transposable elements (987.3 Mb; Table 2), including 491 Mb (19.4%) and 154 Mb (6.1%) by copies of the Gypsy and Copia LTRs, respectively; and 180 Mb (7.1%) by several class 2 DNA transposons (MULE, CMC, Harbinger, etc.)

**Table 2.** Transposable elements identified in the Miscanthus sacchariflorus genome.

Category	Superfamily	Coverage(bp)	Fraction (2.539 Gb)
Class 1 TEs: retrotransposons (copy and paste)	Gypsy LTR	491,915,558	19.37%
	Copia LTR	154,244,411	6.08%
	Other LTRs	87,661,401	3.45%
	SINEs	5,029,476	0.20%
	LINEs	25,076,275	0.99%
	Other non-LTR retrotransposons	29,192,841	1.15%
Class 2 TEs: DNA transposons (cut and paste)	hAT	10,722,644	0.42%
	Harbinger/PIF	24,553,614	0.97%
	MULE/MuDR	29,733,691	1.17%
	Stowaway/TcMar	14,112,359	0.56%
	CMC_EnSpm	56,449,907	2.22%
	Helitron	10,601,152	0.42%
	Other	34,676,501	1.37%
Unclassified TEs	Unclassified TEs	5,934,794	0.23%
Non TEs	Satellites	5,339,464	0.21%
	snRNAs	23,147	0.00%
TOTAL		985,267,235	38.81%

**Figure 1.**
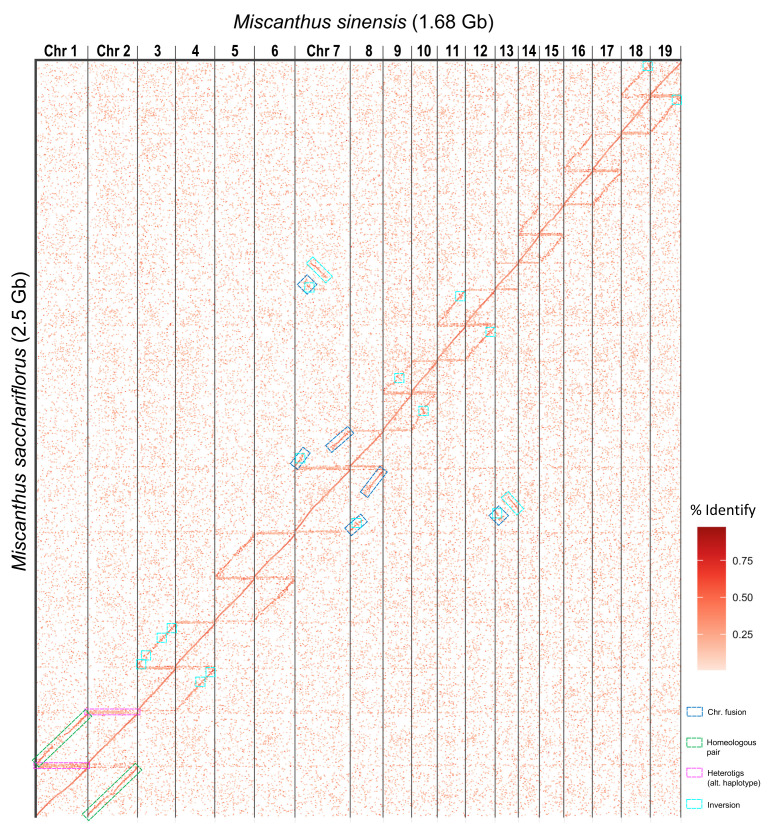
Conserved synteny between *M. sacchariflorus* and *M. sinensis* genomes. The plot shows the primary alignments longer than 2 kbps between both species. The *M. sacchariflorus* scaffolds (Y-axis) have been sorted by their coordinates in *M. sinensis* chromosomes (X-axis). Large homoeologous blocks and chromosomal rearrangements are highlighted in boxes.

We identified 219,394 primary alignments longer than 2 kb between the unanchored *M. sacchariflorus* (“Msac_v2”) and *M. sinensis*. The resulting dotplot ( Figure 1) shows the conserved synteny between both species, which diverged 1.6 Mya ^4^. Figure 1 also shows the highly conserved synteny between the pairs of homoeologous chromosomes (e.g. green boxes in chromosomes one and two), and the fusion in chromosome 7 of the chromosome homeolog to chromosome 13; which was also reported in *M. sinensis* ^4^. There are several large inversions between chromosomes 9 and 10, and 3 and 4 (cyan boxes in Figure 1). Our assembly of a heterozygous genotype resulted in multiple heterotigs (heterozygous contigs) containing the alternative or secondary haplotypes (e.g. pink boxes in Figure 1).

The utility of our assemblies for genomic studies is evidenced by the proportion of RNA-seq from three different tissues from the same *M. sacchariflorus* genotype that aligned to the assemblies. On average 99% and 95% of the RNA-seq reads aligned in “Msac_v2” and “Msac_v3”, respectively ( Table 1).

We estimated that we assembled more than 85% of the *M. sacchariflorus* genes. Furthermore, our assemblies include several alleles of genes in the heterozygous regions of the genome, while the *M. sinensis* reference was generated from a double-haplotyped genotype. The estimation of the proportion of assembled genes (~85%) was supported by (1) the results from BUSCO, which reported 86.4–87.7% of presented core genes, of which ~2/3rds were complete ( Table 1); and (2) the difference in the number of proteins from related species for which we can identify an ortholog in *M. sacchariflorus* compared to *M. sinensis*, as control, using Orthofinder2 ( Table 3).

**Table 3.** Number of orthologs between *Miscanthus sinensis* (Msin), *Setaria italica* (Sita; foxtail millet), *Sorghum bicolor* (Sbic; sorghum), *Zea mays* (Zma; maize), and *Panicum virgatum* (Pvi; switchgrass) obtained using Orthofinder 2.

Orthologs	Msac_v2	Msac_v3	Msin	Sita	Sbic	Zma	Pvi
From Msac_v2 (86,767)	-	NA	44,151 (50.9%)	36,904 (42.5%)	37,219 (42.9%)	38,478 (44.3%)	45,792 (52.8%)
From Msac_v3 (68,578)	NA	-	38,122 (55.6%)	32,273 (47.1%)	32,296 (47.1%)	33,395 (48.7%)	38,755 (56.5%)
From Msin (67,789)	43,739 (64.5%)	37,501 (55%)	-	41,532 (64.1%)	43,475 (64.1%)	39,986 (58.9%)	45,913 (67.7%)
From Sita (40,599)	26,846 (66.1%)	23,559 (58%)	28,473 (70.1%)				
From Sbic (39,441)	27,877 (70.7%)	24,125 (61.2%)	30,907 (78.4%)				
From Zma (88,760)	41,530 (46.8%)	35,955 (40.5%)	41,784 (47.1%)				
From Pvi (125,439)	63,692 (50.8%)	56,120 (44.7%)	64,271 (51.2%)				

Based on the results from Orthofinder2 ( Table 3), we found orthologs in *M. sacchariflorus* for 64.5% of the *M.sinensis* annotated proteins, so we estimated ~1/3 ^rd^ of the Miscanthus proteins to be specific to each species. On the other hand, we estimated that ~3,000 genes may be missing in the “Msac_v2” annotation based on the number of *Sorghum bicolor* proteins with orthologues in *M. sinensis* but absent in *M. sacchariflorus*. Better estimations were obtained with the other four species, where the genes absent in Msac_v2 compared with *M. sinensis* were estimated to be 254, 579 and 1627 ( Table 3). Additionally, ~6,000 genes could be missed in “Msac_v3” compared to “Msac_v2” based on the difference in the number of *M. sinensis* orthologues in each assembly ( Table 3). This is likely from genes in the sequences shorter than 2 Kbps (totalling 402 Mbps) that were filtered out before anchoring. There was a large difference in the proportion of “fragmented” BUSCO genes found in the *M. sacchariflorus* (32.2%) and *M. sinensis* (1.6%) assemblies ( Table 1). To assess if that difference had an effect on the quality of the annotation, we compared the number of proteins from *M. sacchariflorus* and *M. sinensis* for which we can identify an ortholog in another species ( Table 3); we found the difference between both *Miscanthus* species ranged between 6,571 proteins when compared to sorghum (43,475 to 37,219; Table 2) to only 121 when compared to maize (39,986 to 38,478, Table 3).

In conclusion, our *M. sacchariflorus* genome can served as the basis for functional genetic analyses on *Miscanthus,* one of the main biofuel grass crops used in temperate latitudes. However, there are opportunities to improve it using new approaches, such as long-reads.

## Data availability

### Underlying data

NCBI BioProject: Miscanthus sacchariflorus cultivar:Robustus 297. Accession number PRJNA435476; https://identifiers.org/bioproject:PRJNA435476.

This BioProject contains the raw paired-end and mate-pair reads.

NCBI BioProject: RNA-seq Miscanthus hybrids with contrasting phenotypes. Accession number PRJNA639832; https://identifiers.org/bioproject:PRJNA639832.

This BioProject contains RNA-seq reads, deposited as part of a previous project ^29^.

NCBI BioProject: Miscanthus sacchariflorus cultivar:Robustus 297. Accession number PRJNA679435; https://identifiers.org/bioproject:PRJNA679435.

This Bioproject contains the unanchored “Msac_v2” assemblies and gene annotations under accession JADQCR000000000.

The anchored “Msac_v3” assemblies and gene annotations are deposited under accession accession GCA_002993905 under Bioproject PRJNA435476.

The chromosomal positions in the *M. sinensis* chromosomes of the scaffolds from the “Msac_v3” assembly are available in an AGP file as part of GCA_002993905, which places the scaffolds in 19 chromosomes (accessions CM009591 to CM009609 in NCBI).

Zenodo: Supplementary dataset to "Draft genome assembly of the biofuel grass crop Miscanthus sacchariflorus". http://doi.org/10.5281/zenodo.4270235.

This project contains the assemblies in FASTA format, gene annotations in GFF3 format, functional annotations in tabulated text format, and AGP file with anchoring information.

Data deposited with Zenodo are available under the terms of the Creative Commons Attribution 4.0 International license (CC-BY 4.0).
