# Supplementary material for: Draft genome assembly of the biofuel grass crop Miscanthus sacchariflorus
Source: F1000Res. 2021 Jan 18;10:29. [Version 1] doi: 10.12688/f1000research.44714.1 (PMC7921889; doi:10.12688/f1000research.44714.1)
Supplement: Peer review history

# Peer review: Reviewer response for version 1

**DOI:** 10.5256/f1000research.47786.r78040

**Published:** 2021-03-01

**Authors:** Maria Stefanie Dwiyanti

**Affiliations:** 1 Research Faculty of Agriculture, Hokkaido University, Sapporo, Japan

The availability of *Miscanthus sacchariflorus* genome sequence will be useful for *Miscanthus* related research, particularly in bioenergy related topics. 

*"Better estimations were obtained with the other four species, where the genes absent in Msac_v2 compared with M. sinensis were estimated to be 254, 579 and 1627 (Table 3)."*

- I found that the difference between number of genes in *"Msac_v2"* compared to other four species is larger than 254, 579, and 1627; or the way I look into the table is wrong?
- Perhaps the sentence above can be reworded so we can easily compare with the Table 3 content.

Also, what are the predicted functions of genes absent in *"Msac_v2"* compared to *M.sinensis*?

- This information may provide some clues to trait difference between *M. sacchariflorus* and *M.sinensis.*

Are sufficient details of methods and materials provided to allow replication by others?

Yes

Is the rationale for creating the dataset(s) clearly described?

Yes

Are the datasets clearly presented in a useable and accessible format?

Yes

Are the protocols appropriate and is the work technically sound?

Yes

Reviewer Expertise:

Plant genetics and genomics

I confirm that I have read this submission and believe that I have an appropriate level of expertise to confirm that it is of an acceptable scientific standard.

# Peer review: Reviewer response for version 1

**DOI:** 10.5256/f1000research.47786.r77622

**Published:** 2021-02-25

**Authors:** Diego Mauricio Riaño-Pachón

**Affiliations:** 1 Computational, Evolutionary and Systems Biology Laboratory, Center for Nuclear Energy in Agriculture (CENA), University of São Paulo, Piracicaba, Brazil

The Data Note, "Draft genome assembly of the biofuel grass crop *Miscanthus sacchariflorus*", introduces two *Miscanthus sacchariflorus* genome assemblies, which have been deposited in NCBI under the bioprojects: PRJNA679435 and PRJNA435476. Genome sequencing was carried out with Illumina paired end reads and mate-pairs, the assemblies are greatly fragmented, which is expected due to the sequencing technologies used. This is the first *Miscanthus sacchariflorus* genome assembly, which is of interested for the bioenergy community, and can be used to generate insigths with the genomes of other bioenergy crops, like sorghum and sugarcane.

**Suggestions:**

- Look for contaminant organisms in the final assemblies using BlobPlots.
- Provide GenomeScope and Smudgeplots for the clean reads, to generate further statistics prior to assembly.

Are sufficient details of methods and materials provided to allow replication by others?

No

Is the rationale for creating the dataset(s) clearly described?

Yes

Are the datasets clearly presented in a useable and accessible format?

Yes

Are the protocols appropriate and is the work technically sound?

Yes

Reviewer Expertise:

Bioinformatics, genome assembly and annotation.

I confirm that I have read this submission and believe that I have an appropriate level of expertise to confirm that it is of an acceptable scientific standard.
